# Genetic diversity and relationship among indigenous Turkish Karayaka sheep subpopulations

**DOI:** 10.5194/aab-63-269-2020

**Published:** 2020-07-30

**Authors:** Koray Kirikci, M. Akif Cam, Levent Mercan

**Affiliations:** 1 Department of Animal Science, Faculty of Agriculture, Kırşehir Ahi Evran University, 40000, Kırşehir, Turkey; 2 Department of Animal Science, Faculty of Agriculture, Ondokuz Mayıs University, 55200, Samsun, Turkey; 3 Department of Agricultural Biotechnology, Faculty of Agriculture, Ondokuz Mayıs University, 55200, Samsun, Turkey

## Abstract

The Karayaka is the most populous sheep breed in the Black Sea region of Turkey. In the present study, we investigated the intra- and inter-population genetic relationships among indigenous Karayaka sheep subpopulations. Nine microsatellites were genotyped for 64 individuals from Samsun, Ordu, Giresun and Tokat provinces. The average number of alleles ($N_{a}$), allelic richness ($A_{r}$), observed heterozygosity ($H_{o}$), expected heterozygosity ($H_{e}$), polymorphism information content (PIC) and inbreeding coefficient ($F_{\mathrm{IS}}$) for all subpopulations were estimated as $N_{a}=16.44$, $A_{r}=9.887$, $H_{o}=0.303$, $H_{e}=0.886$, PIC=0.866 and $F_{\mathrm{IS}}=0.630$, respectively. The observed and expected heterozygosity ranged from 0.171 (Giresun) to 0.376 (Ordu) and 0.757 (Samsun) to 0.845 (Ordu), respectively. It was determined that a 10.5 % of total genetic variation ($F_{\mathrm{IT}}=66.9$ %) in Karayaka sheep corresponded to genetic differences among subpopulations ($F_{\mathrm{ST}}$), whereas 63.0 % was explained by genetic difference among individuals ($F_{\mathrm{IS}}$). This study gives the first evidence about genetic relationships of Karayaka subpopulations. The results show that Karayaka sheep subpopulations are genetically different from each other. These findings revealed that the Karayaka breed has discrete subpopulations and should be taken into consideration when preparing conservation programs and future breeding strategies.

## 1 Introduction

Karayaka sheep, one of the native Turkish sheep breeds, has been bred under harsh environmental conditions in the Black Sea region of Turkey. Although the history of the Karayaka breed is unknown, the name of “Karayaka” comes from Karayaka village of Tokat province in the Black Sea region (Yalcin, 1986). This breed is distributed along the eastern half of the Black Sea coast, especially in the provinces of Ordu, Giresun, Samsun and Tokat. Karayaka is an important breed for meat production for consumers (Karaman et al., 2013). The sheep population of Turkey was 37 276 050 in 2019 (TUIK, 2019). Karayaka sheep are estimated to constitute 4.5 % of total sheep population in Turkey. Due to its meat quality, Karayaka is highly appreciated and intensively raised in the region.

The conservation of Turkish livestock species has been organized by Turkish Ministry of Agriculture and Forestry with FAO's partnership. Until now, genetic characterization studies on native Turkish sheep breeds and other livestock species have been performed by using microsatellite and other techniques in several studies (Ozmen et al., 2020; Ameur et al., 2020; Kirikci et al., 2018; Ağaoğlu, 2010; Cemal et al., 2013; Das et al., 2015; Elmaci et al., 2007; Kurar et al., 2012; Oner et al., 2013; Ozdemir et al., 2016; Özşensoy, 2011; Yilmaz et al., 2014). These studies provided a reasonable picture of the genetic variation and relationships among native Turkish sheep breeds, but most of those studies were on many breeds.

One of the effective breeding strategies for livestock is to collect extensive knowledge about genetic structure at the population level of a breed (Groeneveld et al., 2010). There has been no study on the genetic relationships among Karayaka sheep subpopulations. Therefore, we aimed to evaluate the genetic relationships within and among Karayaka sheep subpopulations, which has been bred as purebred over the years and reflect the breed's original characteristics.

## 2 Materials and methods

All experimental procedures and animal management were performed under veterinary supervision according to the Animal Care and Use Guidelines of Ondokuz Mayıs University of Local Ethical Committee.

### 2.1 Sampling process

**Figure 1:**
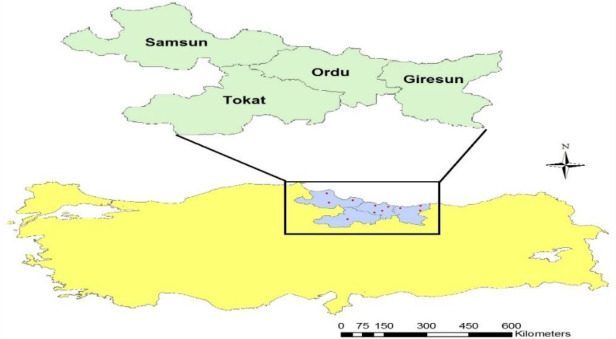
Sampling locations of Karayaka sheep subpopulations.

A total of 64 blood samples were collected from Karayaka sheep populations (16 samples from each population) raised in the provinces of Samsun, Ordu, Giresun and Tokat from the Black Sea region of Turkey. Sampling locations are shown on the map prepared using ArcGIS software (Fig. 1). It was considered that sampled animals were unrelated. In order to make sure, we controlled the pedigree records, stored by the Sheep and Goat Breeders' Association of Turkey. Blood samples were stored at -20 ${}^{\circ }$C until DNA isolations. Genomic DNA was isolated according to the manufacturer's instructions from whole blood using the IDPURE Spin Column Genomic DNA MiniPrep Kit (Empire Genomics, Buffalo, NY).

### 2.2 Microsatellite genotyping

Microsatellites used in the study were selected from the microsatellite list recommended by ISAG/FAOs MoDAD (Measurement of Domestic Animal Diversity) program (FAO, 2004). Microsatellites, their primer sequences and chromosome numbers are given in Table 1. Annealing temperatures (ranged from 55.9 to 63.4 ${}^{\circ }$C) for each locus were determined by gradient PCR method. Polymerase chain reactions were performed in a final volume of 20 µL, including 10 µL of 2X AmpMaster™ Taq (GenALL^®^, South Korea), 1 µL of (10 $\mathrm{pmol}\;µL^{-}$) each primers, 2 µL of genomic DNA (30–50 ng) with ultrapure water added to the final volume. The selected microsatellite loci were amplified using a thermal cycler (Bio-Rad, USA). PCR mixture was heated to 95 ${}^{\circ }$C for 5 min, followed by 35 cycles of, 30 s at a determined annealing temperature for each primer, with a final extension at 72 ${}^{\circ }$C for 4 min. Amplified samples were controlled 2 % agarose gel, staining with EtBr (500 $µL\;{\mathrm{mL}}^{-1}$ in $H_{2}O$). The microsatellite loci studied in the study were genotyped by capillary electrophoresis in Qsep 100™ DNA fragment analyzer (BiOptic Inc., USA) without fluorescently labeled primers. The obtained results were evaluated using BiOptic software provided by the manufacturer.

**Table 1:** Microsatellite marker names, primer sequences, chromosome and GenBank accession numbers.

Marker name	Primer $5^{\prime }$ forward	Primer $3^{\prime }$ reverse	Chromosome	GenBank
			number	
BM757	TGG AAA CAA TGT AAA CCT GGG	TTG AGC CAC CAA GGA ACC	9	G18473.1
BM6526	CAT GCC AAA CAA TAT CCA GC	TGA AGG TAG AGA GCA AGC AGC	19	G18454.1
BM827	GGG CTG GTC GTA TGC TGA G	GTT GGA CTT GCT GAA GTG ACC	3	
BM1314	TTC CTC CTC TTC TCT CCA AAC	ATC TCA AAC GCC AGT GTG G	22	G18433.1
BM8125	CTC TAT CTG TGG AAA AGG TGG G	GGG GGT TAG ACT TCA ACA TAC G	17	G18475.1
CSSM47	TCT CTG TCT CTA TCA CTA TAT GGC	CTG GGC ACC TGA AAC TAT CAT CAT	2	U03821
HUJ616	TTC AAA CTA CAC ATT GAC AGG G	GGA CCT TTG GCA ATG GAA GG	13	M88250.1
MAF33	GAT CTT TGT TTC AAT CTA TTC CAA TTT C	GAT CAT CTG AGT GTG AGT ATA TAC AG	9	M77200.1
OarFCB304	CCC TAG GAG CTT TCA ATA AAG AAT CGG	CGC TGC TGT CAA CTG GGT CAG GG	19	L01535.1

### 2.3 Statistical analysis

The number of alleles, allelic richness, the values of observed heterozygosity ($H_{o}$) and expected heterozygosity ($H_{e}$) for each locus were calculated using FSTAT v.1.2 software package (Goudet, 1995). Wright's F-statistics parameters ($F_{\mathrm{IS}}$ $F_{\mathrm{IT}}$ and $F_{\mathrm{ST}}$) for each population and, in order to control whether Karayaka sheep populations were in Hardy–Weinberg equilibrium, the ARLEQUIN v.3.1. software were used (Schneider et al., 2000). The polymorphic information content values were calculated according to Botstein et al. (1980) for all loci at the population level using CERVUS v.3.0.7 software (Kalinowski et al., 2010). factorial correspondence analysis (FCA) was performed to show the genetic admixture between populations using GENETIX v.4.0.5 software (Belkhir et al., 2004). Genetic differentiation among subpopulations was calculated based on pairwise $F_{\mathrm{ST}}$ values using GENEPOP (Rousset, 2008). Bayesian cluster analysis was performed using the Structure v.2.3.4 software (Pritchard et al., 2000). The probability of an admixture model was calculated for K values ranging from 2 to 5. The results were analyzed with Structure Harvester to generate the ΔK values according to the heuristic method introduced by Earl (2012).

## 3 Results

### 3.1 Genetic diversity parameters

Genetic parameters such as allele number and allelic richness are a good indicator of genetic diversity within populations for studied markers (Hassen et al., 2012). Genetic diversity parameters belong to locus are given in Table 2. In the present study, a total of 148 alleles were observed, with an average number of alleles of 16.44. The highest and lowest number of alleles for all loci was observed 22 at the BM1314 and 8 at the CSSM47 loci. The highest and lowest allele number for populations was observed 87 in Ordu and 65 in Giresun. The average allelic richness was calculated as 9.887. The allelic richness values of the highest and lowest for all loci were detected 12.875 at BM1314 locus and 5.824 at CSSM47 locus (Table 2), while the populations the highest and lowest allelic richness values were 8.644 in Ordu and 6.791 in Giresun (Table 3).

**Table 2:** The genetic diversity parameters for all studied loci.

Locus	$N_{a}$	ASR	$A_{r}$	$H_{o}$	$H_{e}$	PIC
BM757	15	170–208	8.220	0.333	0.847	0.823
BM6526	15	152–186	9.844	0.328	0.903	0.886
BM827	16	210–242	10.271	0.281	0.904	0.889
BM1314	22	134–182	12.875	0.333	0.936	0.923
BM8125	14	104–138	8.177	0.313	0.866	0.843
CSSM47	8	128–172	5.824	0.094	0.761	0.723
HUJ616	20	116–178	10.772	0.387	0.901	0.885
MAF33	18	102–146	11.509	0.468	0.928	0.915
OarFCB304	20	154–122	11.497	0.194	0.924	0.911
Mean	16.44		9.887	0.303	0.886	0.866

Abbreviations: $N_{a}$, number of alleles; ASR, allele size range; $A_{r}$, allelic richness; $H_{o}$, observed heterozygosity; $H_{e}$, expected heterozygosity; PIC, polymorphism information content.

### 3.2 Polymorphism information content (PIC)

The first step of revealing the genetic diversity is to prefer using polymorphic microsatellite markers (Álvarez et al., 2004). The polymorphism information content (PIC) values were calculated for studied markers. The PIC values for all loci ranged between 0.923 at BM1314 loci and 0.723 at CSSM47 loci, with an average of 0.866 in Table 2. The PIC mean values were estimated for Samsun, Ordu, Giresun and Tokat subpopulations as 0.689, 0.827, 0.737 and 0.730, respectively (Table 3). It has been suggested that all loci studied are highly informative because the PIC values to be calculated at the locus are higher than 0.50 (Botstein et al., 1980).

### 3.3 Observed ($H_{o}$) and expected ($H_{e}$) heterozygosity

The observed and expected heterozygosity are the most often used parameters in the studies of population genetics (Nei, 1973). The mean $H_{o}$ and $H_{e}$ values for locus were estimated as 0.303 for $H_{o}$ and 0.886 for $H_{e}$ (Table 2). The observed and expected heterozygosity for the populations ranged from 0.171 in Giresun to 0.376 in Ordu and 0.757 in Samsun to 0.845 in Ordu, respectively (Table 3). In terms of genetic diversity, it was found that the Ordu population had significantly higher heterozygosity than other populations (P<0.05). The highest mean allele number was observed in Ordu (9.67), while the lowest was observed in Samsun (8).

**Table 3:** The genetic diversity of Karayaka sheep subpopulations based on the nine microsatellite markers.

Subpopulation	MNA	NPA	$A_{r}$	$H_{o}$	$H_{e}$	$F_{\mathrm{IS}}$	PIC
Samsun	8	16	6.994	0.329	0.757	0.568	0.689
Ordu	9.67	16	8.644	0.376	0.845	0.577	0.827
Giresun	7.22	11	6.791	0.171	0.765	0.791	0.737
Tokat	8.22	10	7.252	0.329	0.775	0.591	0.730

Abbreviations: MNA, mean allele number; NPA, private allele number; $A_{r}$, allelic richness; $H_{o}$, observed heterozygosity; $H_{e}$; expected heterozygosity; $F_{\mathrm{IS}}$, inbreeding coefficient; PIC, polymorphism information content.

The private allele number was calculated comparing populations (Table 3). The numbers of private alleles for both locus and population are given in Table S1 in the Supplement. We observed a total of 53 private alleles, 28 of which had a frequency over 5 %. The highest private allele number (11) was observed in HUJ616 and the lowest (1) in CSSM47. The highest private allele number (16) was observed in Samsun and Ordu, whereas the lowest (10) was observed in Tokat.

### 3.4 Wright's F-statistics parameters ($F_{\mathrm{IS}}$, $F_{\mathrm{IT}}$ and $F_{\mathrm{ST}}$)

Wright's F-statistics parameters calculated for studied loci in Karayaka sheep breed are given in Table 4. It was determined that 10.5 % of the total genetic variation ($F_{\mathrm{IT}}=66.9$ %) in Karayaka sheep corresponded to genetic differences among populations ($F_{\mathrm{ST}}$), whereas the genetic difference explained 63.0 % among individuals ($F_{\mathrm{IS}}$). The $F_{\mathrm{IS}}$ values that were calculated at subpopulation level ranged from 0.568 in Samsun to 0.791 in Giresun, and they were all significant (P<0.001) (Table 4).

**Table 4:** Wright's F-statistics parameters for studied loci.

Locus	$F_{\mathrm{IS}}$	$F_{\mathrm{ST}}$	$F_{\mathrm{IT}}$
BM757	0.642	0.091	0.674
BM6526	0.632	0.111	0.673
BM827	0.625	0.110	0.666
BM1314	0.630	0.112	0.672
BM8125	0.632	0.109	0.672
CSSM47	0.608	0.098	0.646
HUJ616	0.642	0.107	0.681
MAF33	0.654	0.106	0.690
OarFCB304	0.611	0.102	0.651
Mean	0.630±0.039	0.105±0.019	0.669±0.037

### 3.5 Factorial correspondence analysis (FCA)

**Figure 2:**
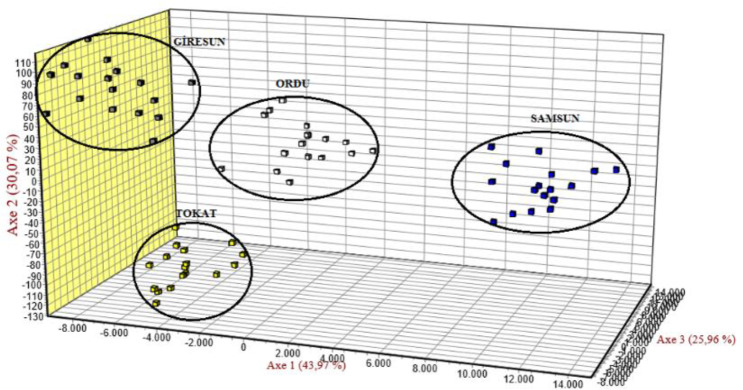
FCA results showing the relationship between the subpopulations.

Genetic similarities for Karayaka sheep subpopulations were revealed with FCA. All the subpopulations were separated from each other, whereas Samsun subpopulation was clustered in the different place than other subpopulations (Fig. 2).

### 3.6 Genetic structure

**Figure 3:**
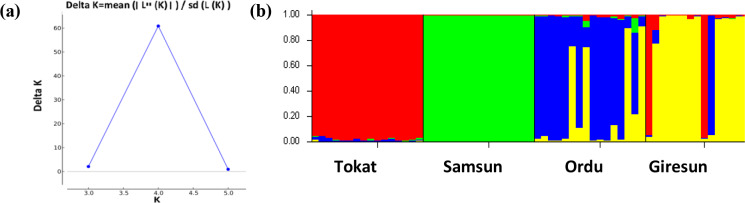
The results of the ΔK **(a)** and Structure analysis **(b)**.

To evaluate the genetic structure and level of admixture among the studied subpopulations, the most likely K value was calculated using the Bayesian approach. In this analysis, the highest ΔK value was K=4. The studied populations were grouped into the four populations (Fig. 3), and this result was in accordance with the FCA result (Fig. 2).

### 3.7 Genetic differentiation between populations 

Genetic differentiation between the populations were compared on the basis of pairwise $F_{\mathrm{ST}}$ (Table 5). $F_{\mathrm{ST}}$ values ranged from 0.061 (Ordu and Tokat) to 0.168 (Giresun and Samsun). Ordu and Tokat were the most similar subpopulations (0.061) whereas the furthest was Samsun and Giresun (0.168) (Table 5). All calculated pairwise $F_{\mathrm{ST}}$ values were significant (0.05), indicating that subpopulations could be genetically different from each other.

**Table 5:** Pairwise $F_{\mathrm{ST}}$ values among subpopulations.

Subpopulation	Tokat	Samsun	Ordu
Samsun	0.152	0.000	0.000
Ordu	0.061	0.090	0.000
Giresun	0.095	0.168	0.068

## 4 Discussion

There are several studies that are interested in the genetic diversity of different sheep breeds in Turkey (Gutierrrez-Gil et al., 2006; Koban, 2004; Yildiran and Cakir, 2012; Yilmaz et al., 2014). In the previous studies of Karayaka breed in literature the numbers of samples were too low. Moreover, there was not another study investigating the Karayaka breed subpopulations. Therefore, we aimed to investigate the genetic diversity and relationships in Karayaka sheep subpopulations by collecting more samples from geographically distinct populations.

The used markers in this study were highly polymorphic because of the PIC values were higher than 0.50 (Botstein et al., 1980). The PIC mean value for the studied loci was 0.866, ranged from 0.723 (CSSM47) to 0.923 (BM1314). This means that all studied markers are highly polymorphic. The average value of PIC was higher than values obtained from the other sheep breeds (Dossybayev et al., 2019; Guang-Xin et al., 2019). The mean number of alleles was 16.44 with a total of 148. The values obtained were higher than those of some previous studies with Asia and Europe sheep breeds (Ceccobelli et al., 2009; Khaleel et al., 2018). Although the mean number of alleles obtained was higher than those by Yilmaz et al. (2014), it was lower than those obtained by Yildiran and Cakir (2012). This difference could be explained by the number of the used loci and different breeds. In the present study, the microsatellites with the highest and lowest allele number were BM1314 (22) and CSSM47 (8), respectively. Similar results were reported by other studies (ranged from 8 to 12 in BM1314 and from 3 to 4 in CSSM47) (Arora and Bhatia, 2004; Singh et al., 2015; Girish et al., 2007).

In the present study, the mean value of expected heterozygosity ($H_{e}$: 0.886) was higher ($H_{e}$: 0.62–0.786) than those reported from previous studies (Selvam and Kathiravan, 2019; Bravo et al., 2019; Girish et al., 2007; Arora et al., 2011; Singh et al., 2015; Lasagna et al., 2011; Jyotsana et al., 2010). These results are probably due to the differences in sampling populations in terms of the population's breeding strategy and region's climatic conditions. Thus, adaptation may have caused this allelic richness and diversity. Also, sampling of unique genotypes and populations could be another possible reason. As the calculated Wright's F-statistics parameters showed in Table 4, the main genetic diversity (66.9 %) resulted from those within subpopulations. The lowest diversity (10.5 %) was found between the subpopulations and was significantly important (P<0.05). The $F_{\mathrm{IS}}$ values that were calculated at subpopulation level ranged from 0.568 (Samsun) to 0.791 (Giresun), and they were all significant (P<0.001). For this reason, subpopulations were not in Hardy–Weinberg equilibrium. The calculated $F_{\mathrm{IS}}$ values for Karayaka sheep were the positive and higher than those of calculated by Yilmaz et al. (2014), Hoda and Marsan (2012), and Gutierrrez-Gil et al. (2006). The positive or high $F_{\mathrm{IS}}$ values could be result in uncontrolled breeding, low herd size and some conditions that increases the inbreeding (Singh et al., 2015; Crispim et al., 2014; Gaouar et al., 2016).

$F_{\mathrm{ST}}$ values varied from 0.061 to 0.168. Genetic differentiation for the studied populations was moderate ($F_{\mathrm{ST}}>0.05$) except for Samsun and Giresun. A high genetic differentiation ($F_{\mathrm{ST}}>0.15$) was observed between Samsun and Giresun. The results of FCA and Structure analyses showed the same findings. Overall, studied populations were grouped separately. The presence of high genetic differentiation among subpopulations is quite interesting when considered for only one breed. These results may be caused by inbreeding and geographic distances among subpopulations. Another reason for this may be that these populations are pretty much isolated and small (Al-Atiyat, 2016).

Results of both FCA and Structure for the Ordu and Giresun subpopulations were more similar than the rest of the other subpopulations. This result could be attributed to the fact that the animals shared the same areas in the grazing season. The existence of genetic variability and distinction among populations could have important consequences for maintaining genetic diversity, planning of breeding programs and conservation studies in the future (Wu et al., 2015). The high genetic diversity within a population and differentiation among populations could be due to either geographic distance or allelic richness and other factors.

## 5 Conclusion

Conservation studies in livestock should be done at the population levels of a specific breed. Because this provides comprehensive knowledge of the genetic diversity and genetic structure among and within populations and possibilities for determination of conservation strategies. We studied isolated populations consisting of a low number and pure breeding. This kind of population could be more vulnerable to genetic drifts. This work provides the first evidence for genetic relationships among Karayaka sheep subpopulations. Consequently, Karayaka sheep subpopulations were genetically different from each other in terms of nine microsatellite loci, meaning that this breed has discrete subpopulations; this should be taken into consideration when preparing conservation programs and future breeding strategies.

## Supplement

10.5194/aab-63-269-2020-supplement The supplement related to this article is available online at: https://doi.org/10.5194/aab-63-269-2020-supplement.

## Data Availability

The data sets are available upon request from the corresponding author.
