# Supplementary material for: Genetic diversity and relationship among indigenous Turkish Karayaka sheep subpopulations
Source: Arch Anim Breed. 2020 Jul 30;63(2):269–75. doi: 10.5194/aab-63-269-2020 (PMC7405648; doi:10.5194/aab-63-269-2020)
Supplement: The supplement related to this article is available online at: https://doi.org/10.5194/aab-63-269-2020-supplement. [file aab-63-269-supplement.pdf]

Supplement of Arch. Anim. Breed., 63, 269–275, 2020
https://doi.org/10.5194/aab-63-269-2020-supplement
© Author(s) 2020. This work is distributed under
the Creative Commons Attribution 4.0 License.

Open Access
Archives Animal Breeding

Supplement of

Genetic diversity and relationship among indigenous Turkish Karayaka sheep subpopulations

Koray Kirikci et al.

Correspondence to: Koray Kirikci (koray.kirikci@ahievran.edu.tr)

The copyright of individual parts of the supplement might differ from the CC BY 4.0 License.

Table S1. Private alleles and their frequencies for loci per population.

Locus name	Alleles	Populations			
		Samsun	Ordu	Giresun	Tokat
BM757	170			0.067*	
	174			0.133*	
	178		0.031		
	180		0.219*		
	192		0.031		
	194	0.031			
	196	0.031			
	208	0.031			
BM827	210	0.031			
	214	0.063*			
	220			0.252*	
	238				0.063*
	242	0.031			
BM6526	152			0.071*	
	156			0.071*	
	160				0.031
	182	0.031			
	186	0.033			
BM8125	104	0.031			
	108			0.031	
	110				0.031
BM1314	134		0.071*		
	136		0.036		
	138				0.031
	140	0.077*			
	146			0.273*	
	150				0.094*
	162				0.125*
	182	0.077*			
CSSM47	140			0.063*	
HUJ616	116			0.143*	
	118		0.188*		
	120		0.094*		
	130	0.125*			
	134		0.063*		
	142	0.031			
	148			0.071*	
	150	0.031			
	160				0.063*
	166		0.063*		
	178		0.063*		
MAF33	102		0.031		
	112		0.063*		
	114		0.031		
	116			0.094*	
	130		0.063*		
	140	0.333			
	146	0.033			
OarFCB304	164		0.031		
	180		0.031		
	186				0.154*
	210				0.039
	222				0.039
Total		16	16	11	10

*Private alleles with frequency higher than 5%.
